# A novel compound heterozygous mutation in *TREM2* found in a Turkish frontotemporal dementia-like family^☆^

**DOI:** 10.1016/j.neurobiolaging.2013.06.005

**Published:** 2013-12

**Authors:** Rita Guerreiro, Basar Bilgic, Gamze Guven, José Brás, Jonathan Rohrer, Ebba Lohmann, Hasmet Hanagasi, Hakan Gurvit, Murat Emre

**Affiliations:** a Department of Molecular Neuroscience, Institute of Neurology, University College London, London, UK; b Behavioral Neurology and Movement Disorders Unit, Department of Neurology, Istanbul Faculty of Medicine, Istanbul University, Istanbul, Turkey; c Genetics Department, Institute for Experimental Medicine, Istanbul University, Istanbul, Turkey; d Dementia Research Centre, Institute of Neurology, University College London, London, UK; e Department of Neurodegenerative Diseases, Hertie Institute for Clinical Brain Research, University of Tübingen and German Center for Neurodegenerative Diseases (DZNE), Tübingen, Germany

**Keywords:** *TREM2*, Nasu-Hakola, Frontotemporal dementia, Compound heterozygous

## Abstract

Triggering receptor expressed on myeloid cells 2 (*TREM2*) homozygous mutations cause Nasu-Hakola disease, an early-onset recessive form of dementia preceded by bone cysts and fractures. The same type of mutations has recently been shown to cause frontotemporal dementia (FTD) without the presence of any bone phenotype. Here, we further confirm the association of *TREM2* mutations with FTD-like phenotypes by reporting the first compound heterozygous mutation in a Turkish family.

## 1 Introduction

Nasu-Hakola disease (NHD, OMIM 221770), also known as polycystic lipomembranous osteodysplasia with sclerosing leukoencephalopathy, is a rare autosomal recessive disease characterized by a combination of progressive young-onset dementia and multifocal bone cysts (Paloneva et al., 2001). In most Nasu-Hakola patients, the clinical course of the disease can be divided into 4 stages: (1) the latent stage with normal early development; (2) the osseous stage beginning at the third decade of life, characterized by pain and swelling in ankles and feet followed by frequent bone fractures; (3) the early neuropsychiatric stage occurring at the fourth decade of life, presenting with a frontal lobe syndrome including euphoria and social disinhibition; and (4) the late neuropsychiatric stage, characterized by profound dementia, loss of mobility, and death usually by age of 50 years (Klunemann et al., 2005). NHD is caused by mutations in 1 of 2 genes: TYRO protein tyrosine kinase–binding protein (*TYROBP*) on chromosome 19q13.1 and triggering receptor expressed on myeloid cells 2 (*TREM2*) on chromosome 6p21.1.

Recently, cases with homozygous mutations in *TREM2* have been described to present atypical phenotypes resembling behavioral variant frontotemporal dementia (bvFTD) and lacking stage (2) of the clinical picture described previously (Chouery et al., 2008; Giraldo et al., 2013; Guerreiro et al., 2013b).

At present, 14 different mutations have been identified in *TREM2* including nonsense, missense, small deletions, and splice site homozygous mutations (Giraldo et al., 2013). Here, we report the first *TREM2* compound heterozygous mutation identified in a Turkish family presenting with an FTD-like phenotype.

## 2 Methods

### 2.1 Patients

The proband is currently being followed in the outpatient clinic of the Behavioral Neurology and Movement Disorders Unit, Istanbul Faculty of Medicine, Istanbul University. She was hospitalized in 2012 for a detailed neurologic workup. Written informed consent was obtained from all family members, and this study was approved by the ethical committee of the Istanbul University.

### 2.2 Genetic analysis

DNA was extracted from blood samples, according to standard procedures. For the proband, all coding exons of *TREM2* were amplified by polymerase chain reaction (PCR) using Roche FastStart PCR Master Mix (Roche Diagnostics Corp). The PCR products were sequenced using the same forward and reverse primers (primers available on request) with Applied Biosystems BigDye terminator version 3.1 sequencing chemistry and run on an ABI3730XL genetic analyzer as per the manufacturer's instructions (Applied Biosystems). The sequences were analyzed using Sequencher software, version 4.2 (Gene Codes). For family members, only exon 2 of *TREM2* was tested.

## 3 Results

### 3.1 Case report

The proband is a 41-year-old female who presented with a 1-year history of a change in personality. She had become increasingly apathetic, and her husband reported that she had become less empathic toward close family and friends, unexpectedly leaving her husband and their child in the United States to move back to her parents' house in Turkey. At this time, her mother noticed that she had started to become more perseverative in her behavior and developing a change in food preference, eating more junk food. She also started to have mild word-finding difficulties. She had been previously fit and well apart from having had 2 previous generalized tonic-clonic seizures (one 4 years earlier and one 2 years earlier).

**Fig. 1:**
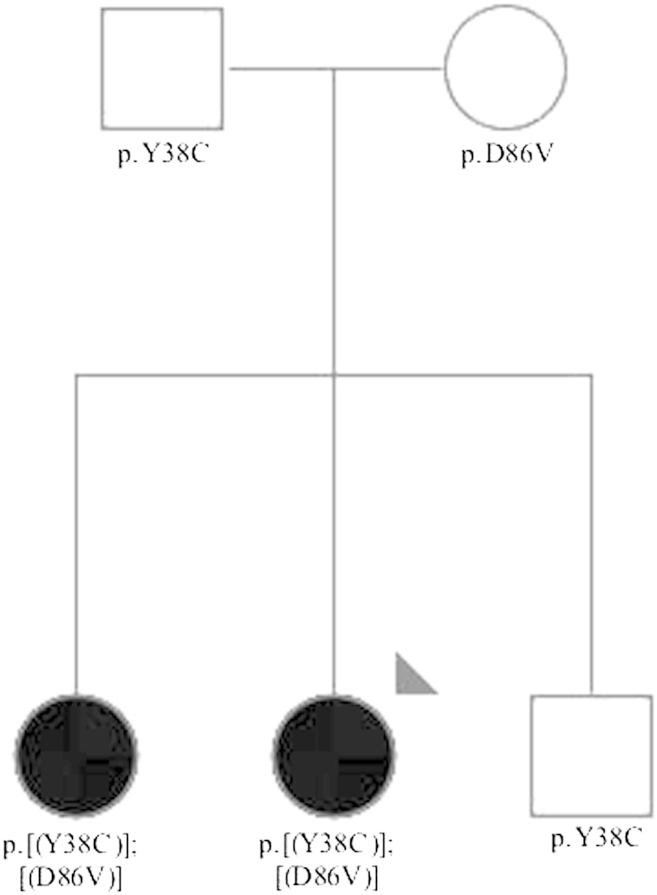
Pedigree of the family where the compound heterozygous mutation p.[(Y38C)];[(D86V)] in *TREM2* was found. The arrowhead indicates the proband. Black filled symbols represent affected subjects with dementia. White symbols represent unaffected family members. The *TREM2* variants found are represented below each individual showing segregation of the compound heterozygous mutation with the disease.

Her parents were originally from the same city (Erzincan) with a population of about 225,000 inhabitants, located in the eastern part of Turkey and were both clinically healthy. She had 2 siblings, a brother who was well and a sister who had also developed progressive cognitive impairment and behavioral problems with generalized tonic-clonic seizures starting in her 30s (Fig. 1).

She scored 44 on the Addenbrooke's cognitive examination (range 0–100, higher scores indicating better performance) and 28 on the frontal behavioral inventory (range 0–72, higher scores indicating worse behavioral symptoms; disinhibition subscore 11, negative behavior subscale score 17). Formal neuropsychometry testing revealed deficits in executive function, particularly abstract thinking, planning, and inhibition. There was also evidence of language dysfunction with nonfluent speech, anomia, and phonemic paraphasias although comprehension and repetition were normal. Other cognitive domains were relatively intact, and both neurologic and general systemic examinations were normal.

**Fig. 2:**
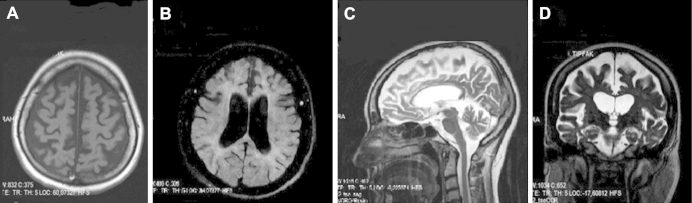
Magnetic resonance imaging of the proband. (A) Transverse T1-weighted image shows atrophy of the frontoparietal cortices. (B) Transverse fluid-attenuated inversion recovery image demonstrates mild but diffuse hyperintense appearance of the white matter, mainly located in the neighborhood of the ventricle horns. (C) In sagittal T2-weighted image, there is marked thinning of the entire corpus callosum. (D) Coronal T2-weighted image reveals enlargement of the lateral and third ventricles with atrophy of the bilateral caudate nuclei.

**Fig. 3:**
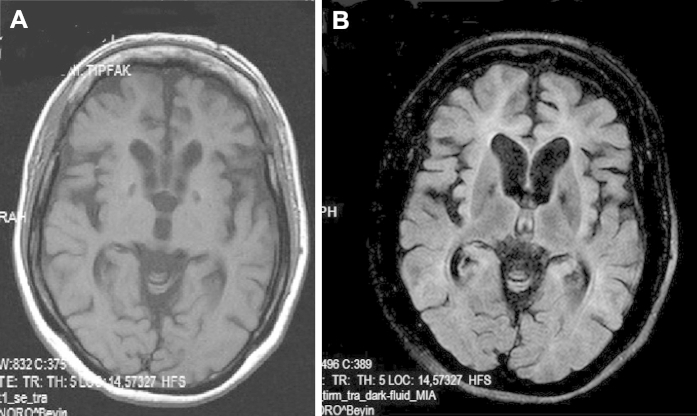
Both T1 and fluid-attenuated inversion recovery images demonstrate symmetrical hypointense lesions in basal ganglia.

**Fig. 4:**
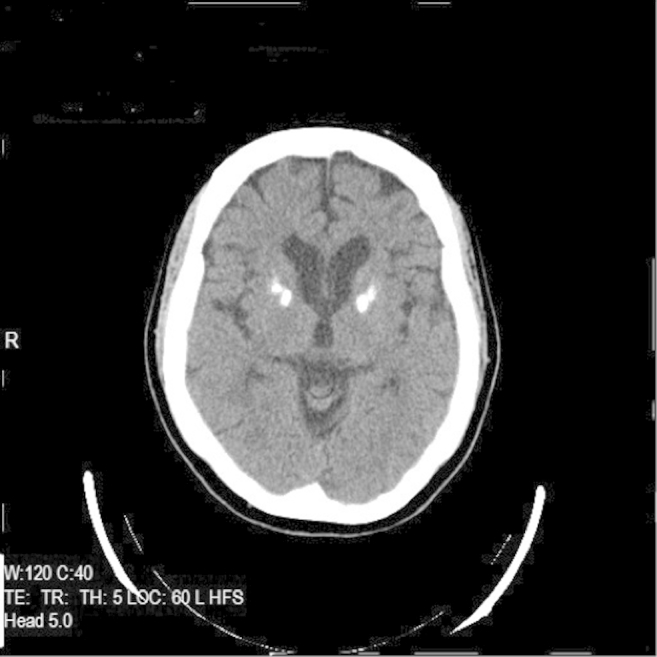
Computed tomography of the proband reveals symmetrical hyperdense lesions resembling calcifications in the globus pallidus.

Blood tests were normal including serum calcium, phosphate, and parathyroid hormone. Cerebrospinal fluid analysis revealed a mild pleocytosis (10 lymphocytes per cubic millimeter) and mildly increased protein (54 mg/dL) with positive unmatched oligoclonal bands. Electroencephalography was normal. Magnetic resonance imaging of the brain showed atrophy mainly located in the frontal, lateral temporal, and parietal cortices and also the caudate nuclei with marked thinning of the corpus callosum and enlargement of the ventricular system. On T2 and fluid-attenuated inversion recovery imaging, there was evidence of diffuse hyperintensities in the periventricular, frontal, and occipitoparietal white matter (Fig. 2). There were also symmetrical hypointense lesions bilaterally in the globus pallidus on T1, T2, and fluid-attenuated inversion recovery imaging (Fig. 3). These were considered to be calcification because computed tomography of the brain revealed marked hyperdense lesions (Fig. 4) in the same areas. Further tests included a skeletal X-ray survey, which showed no bone cysts.

### 3.2 Genetic analysis

In the proband, PCR and direct sequencing analysis of the 5 exons and exon-intron boundaries of *TREM2* identified 2 different missense heterozygous mutations: c.113A>G, p.Y38C and c.257A>T, p.D86V in exon 2 (NM_018965.3).

To determine if the patient was a compound heterozygote, we sequenced exon 2 in the parents. The father carried the p.Y38C heterozygous variant and the mother carried the p.D86V also heterozygous; thus, the proband carries a compound heterozygous mutation: c.[113A>G];[257A>T]; p.[(Y38C)];[(D86V)].

To assess segregation of the mutation with the disease, we sequenced 2 siblings, 1 affected and 1 unaffected. The compound heterozygous mutation segregated with the disease, being present in the affected siblings and absent in the unaffected, who carried only one of the variants (p.Y38C) (Fig. 1).

None of these variants are reported on large genetic databases (dbSNP version 137, 1000Genomes Project or Exome Variant Server, NHLBI GO Exome Sequencing Project, Seattle, WA, http://evs.gs.washington.edu/EVS/, accessed on May, 2013), but we have previously reported both changes in the Turkish population. Homozygosity for the p.Y38C mutation was found in the only affected subject from family 2, and p.D86V was found in heterozygosity in a healthy control individual from 76 Turkish controls screened for *TREM2* exon 2 mutations in our previous study (Guerreiro et al., 2013b).

## 4 Discussion

Both homozygous and compound heterozygous mutations in *TYROBP* are known to cause NHD (Kuroda et al., 2007). Homozygous mutations in *TREM2* also cause NHD. More recently, we and others have found homozygous mutations in this gene to be associated with bvFTD-like phenotype without any associated bone involvement (Chouery et al., 2008; Giraldo et al., 2013; Guerreiro et al., 2013b). Here, we report the first compound heterozygous mutation in *TREM2*, which was found to be associated with FTD phenotype in a Turkish family.

The proband in the family here described was identified because she presented a clinical picture similar to the 3 previous bvFTD-like cases with homozygous *TREM2* mutations we reported before (Guerreiro et al., 2013b). In addition to these 3 Turkish cases, 2 other families were also reported to carry homozygous mutations in *TREM2*, presenting with a bvFTD-like phenotype without bone cysts. Chouery et al. (2008) described a Lebanese family presenting with a young-onset dementia without bone cysts where 3 affected subjects carried a deletion (c.40+3delAGG) in the 5′ consensus donor splice site in intron 1 of *TREM2*. These affected individuals developed personality and behavioral changes at the age of 30–35 years, culminating in severe dementia. Brain magnetic resonance imaging showed cortical atrophy with periventricular white matter disease (Chouery et al., 2008). More recently, Giraldo et al. (2013) reported a novel homozygous nonsense mutation (p.W198X) in a Colombian family with an autosomal recessive pattern of inheritance of FTD, also without bone cysts. These patients started to develop behavioral and personality changes around the age of 45–50 years. Similar to this case and the previously described Turkish cases, the affected siblings in the Colombian family also developed seizures (Table 1).

Dementia in the form of an early-onset personality change, resembling bvFTD, seems to be the most common feature of all these reported cases, most often associated with seizures and characteristic imaging findings, such as callosal atrophy and diffuse white matter lucency. Familial FTD accounts for >40% of all cases, and to date, several genetic causes of the phenotype have been described. However, all the 7 genes with mutations causing familial FTD (i.e., *MAPT*, *GRN*, *C9ORF72*, *VCP*, *CHMP2B*, *TARDBP*, and *FUS*) are dominantly inherited. *TREM2* mutations appear to be the first addition to this list as a recessively inherited genetic cause. We suggest that a family history of consanguinity along with somewhat atypical features, such as seizures and callosal atrophy, in a patient presenting with a bvFTD phenotype should prompt the diagnostician to look for a likely *TREM2* homozygous or compound heterozygous mutation. In the same line of reasoning, this genetic cause should be included in the differential diagnosis of young-onset dementia with seizures. Hitherto, adult neuronal ceroid lipofuscinosis (also known as Kufs disease) remains the prototype of this presentation.

The precise molecular and cellular mechanisms underlying the pathogenesis of these diseases are not yet known. There seems to be no correlation between the type of mutation and the associated phenotype because nonsense, splice site, and missense mutations have now been associated with FTD cases. TREM2 is a membrane protein that forms a receptor-signaling complex with TYROBP, triggering the activation of the immune response, differentiation of dendritic cells and osteoclasts, and phagocytosis in microglia. It is interesting to note that a heterozygous variant (p.R47H) in *TREM2* has recently been shown to increase the risk for Alzheimer's disease (Guerreiro et al., 2013a; Jonsson et al., 2013). This suggests that TREM2 is part of a functional network involved in different neurodegenerative dementias (FTD, Alzheimer's disease, and Nasu-Hakola), but besides the fact that homozygous mutations cause an early-onset and more severe disease, whereas heterozygous variants increase the risk for a late-onset disorder, it is not possible to establish a specific correlation between genotype and phenotype.

## Disclosure statement

The authors have no actual or potential conflicts of interest.

## Figures and Tables

**Table 1:** Main features of all the patients described in the literature with *TREM2* mutations presenting without an osseous phenotype

	Chouery et al. (2008)			Guerreiro et al. (2013b)			Giraldo et al. (2013)				Present study	
Number of families	1			3			1				1	
Geographic origin	Lebanon			Turkey			Colombia				Turkey	
	Sibling 1	Sibling 2	Sibling 3	Patient 1	Patient 2	Patient 3	Sibling 1		Sibling 2	Sibling 3 (index)	Sibling 1	Sibling 2 (index)
Gender	F	F	M	M	M	M	M		F	F	F	F
Diagnosis	Early-onset dementia			bvFTD like			bvFTD				bvFTD like	
Age at onset (y)	30–35			20	Late 30s	33	50	45		47	32	36
Age at death (y)	50	Alive (50)	46	33	Alive (50)	45	Alive (55)	NA		Alive (48)	Alive (40)	Alive (41)
Initial symptoms	Forgetfulness and fatigue			Personality changes with aggressive behavior	Personality changes with aggressive and perseverative behavior	Generalized T-C seizure	Altered social behavior and oropharyngeal tic in 1 sibling				Behavioral changes with nonfluent aphasia	Generalized T-C seizures
Other symptoms	Neuropsychiatric signs UI and impotence			Generalized T-C seizures Cognitive impairment Ophthalmoplegia with bradykinesia and brisk deep tendon reflexes	Cognitive impairment Bradykinesia, apraxia + postural instability	Personality changes with progressive behavioral problems Bradykinesia + mild postural instability Visual hallucinations UI	New onset of substance use Complex partial seizures Severe pan-frontal syndrome and cognitive decline				Generalized T-C seizures Bradykinesia + postural instability UI	Cognitive deterioration + behavioral changes
Brain imaging	Diffuse brain and cortical atrophy in the polar region of the temporal lobes CC thinning Stenosis of the aqueduct of sylvius + arachnoid cyst in the posterior cranial fossa Periventricular leucoaraiosis Relative enlargement of perivascular Virchow-Robin spaces No lenticulopallidal calcifications			Frontal and temporal cortical atrophy CC thinning Diffuse confluent WM abnormalities No calcification of the basal ganglia	Frontal cortical atrophy with marked ventricular enlargement CC thinning Confluent periventricular WM abnormalities	Cortical atrophy predominantly affecting the frontal loes Diffuse confluent WM lesions No calcification of the basal ganglia	Asymmetric bifrontal atrophy				Frontoparietal and temporal cortical atrophy Marked CC thinning Diffuse periventricular and frontal WM lesions No calcification of the basal ganglia	Global cortical atrophy mainly in frontal, lateral temporal and parietal cortices and caudate nuclei CC thinning Enlargement of ventricular system Hyperintensity in periventricular, frontal and occipitoparietal WM Bilateral calcification in globus pallidus
Family history	No history of dementia in the parents			History of dementia, psychotic disorders, and epilepsy in the family	One brother, paternal uncle, and maternal grandfather presented late-onset memory impairment	No family history of cognitive or behavioral impairment	One paternal uncle and brother had similar symptoms				No family history of cognitive or behavioral impairment	
TREM2 mutation	c.40+3delAGG			c.97C>T; p.Q33X	c.197C>T; p.T66M	c.113A>G; p.Y38C	c.594G>A; p.W198X				c.[113A>G];[257A>T]; p.[(Y38C)];[(D86V)]	

Key: bvFTD, behavioral variant frontotemporal dementia; CC, corpus callosum; F, female; M, male; NA, not applicable; T-C, tonic-clonic; UI, urinary incontinence; WM, white matter.
